# A biallelic mutation in *IL6ST* encoding the GP130 co-receptor causes immunodeficiency and craniosynostosis

**DOI:** 10.1084/jem.20161810

**Published:** 2017-09-04

**Authors:** Tobias Schwerd, Stephen R.F. Twigg, Dominik Aschenbrenner, Santiago Manrique, Kerry A. Miller, Indira B. Taylor, Melania Capitani, Simon J. McGowan, Elizabeth Sweeney, Astrid Weber, Liye Chen, Paul Bowness, Andrew Riordan, Andrew Cant, Alexandra F. Freeman, Joshua D. Milner, Steven M. Holland, Natalie Frede, Miryam Müller, Dirk Schmidt-Arras, Bodo Grimbacher, Steven A. Wall, E. Yvonne Jones, Andrew O.M. Wilkie, Holm H. Uhlig

**Affiliations:** 1 Translational Gastroenterology Unit, John Radcliffe Hospital, University of Oxford, Oxford, England, UK; 2 Clinical Genetics Group, MRC Weatherall Institute of Molecular Medicine, John Radcliffe Hospital, University of Oxford, Oxford, England, UK; 3 Division of Structural Biology, Wellcome Trust Centre for Human Genetics, University of Oxford, Oxford, England, UK; 4 Computational Biology Research Group, MRC Weatherall Institute of Molecular Medicine, John Radcliffe Hospital, University of Oxford, Oxford, England, UK; 5 Nuffield Department of Orthopaedics, Rheumatology, and Musculoskeletal Sciences, University of Oxford, Oxford, England, UK; 6 Craniofacial Unit, Department of Plastic and Reconstructive Surgery, Oxford University Hospitals National Health Service Foundation Trust, John Radcliffe Hospital, University of Oxford, Oxford, England, UK; 7 Department of Paediatrics, University of Oxford, Oxford, England, UK; 8 Dr. von Hauner Children's Hospital, Ludwig-Maximilians-University of Munich, Munich, Germany; 9 Department of Clinical Genetics, Liverpool Women's National Health Service Foundation Trust, Liverpool, England, UK; 10 Department of Paediatric Infectious Diseases and Immunology, Alder Hey Children's National Health Service Foundation Trust, Liverpool, England, UK; 11 Institute of Cellular Medicine, Newcastle University, Newcastle upon Tyne, England, UK; 12 Laboratory of Clinical Infectious Diseases, National Institute of Allergy and Infectious Diseases, National Institutes of Health, Bethesda, MD; 13 Laboratory of Allergic Diseases, National Institute of Allergy and Infectious Diseases, National Institutes of Health, Bethesda, MD; 14 Center for Chronic Immunodeficiency, Universitätsklinikum Freiburg, Freiburg, Germany; 15 Inflammation and Cancer Lab, Institute of Biochemistry, Christian-Albrechts-University Kiel, Kiel, Germany; 16 Institute of Immunology and Transplantation, Royal Free Hospital, University College London, London, England, UK

## Abstract

Multiple cytokines, including interleukin 6 (IL-6), IL-11, IL-27, oncostatin M (OSM), and leukemia inhibitory factor (LIF), signal via the common GP130 cytokine receptor subunit. In this study, we describe a patient with a homozygous mutation of *IL6ST* (encoding GP130 p.N404Y) who presented with recurrent infections, eczema, bronchiectasis, high IgE, eosinophilia, defective B cell memory, and an impaired acute-phase response, as well as skeletal abnormalities including craniosynostosis. The p.N404Y missense substitution is associated with loss of IL-6, IL-11, IL-27, and OSM signaling but a largely intact LIF response. This study identifies a novel immunodeficiency with phenotypic similarities to STAT3 hyper-IgE syndrome caused by loss of function of GP130.

## Abstract (precis)

Schwerd et al. report a novel homozygous missense substitution in the cytokine co-receptor GP130 encoded by *IL6ST*. This is associated with defective IL-6, IL-11, OSM, and IL-27 signaling and causes immunodeficiency and skeletal abnormalities with similarities to STAT3 hyper-IgE syndrome.

## Introduction

The evolutionarily conserved GP130 cytokine receptor superfamily mediates interactions between a wide range of immune and nonimmune cells (Sims, 2009; O’Shea and Plenge, 2012). GP130 can bind several distinct receptor subunits such as IL-6RΑ, IL-11RA, leukemia inhibitory factor (LIF) receptor, oncostatin M (OSM) receptor, or ciliary neurotrophic factor (CNTF) receptor, facilitating recognition of multiple ligands including IL-6, IL-11, IL-27, LIF, OSM, CNTF, cardiotrophin 1 (CT1), and cardiotrophin-like cytokine (CLC). Signal transduction via GP130 is mediated by the JAK/STAT pathway and includes phosphorylation of STAT3 and STAT1, as well as activation of RAS/MAPK (O’Shea and Plenge, 2012).

An essential role for GP130-dependent signaling is shown by the lethality of the corresponding homozygous KO (*Il6st^−/−^*) in mice, caused by myocardial and hematological defects (Yoshida et al., 1996). In addition, GP130-deficient mice have multiple skeletal abnormalities because of a failure in osteoblast and osteoclast function (Kawasaki et al., 1997; Shin et al., 2004). Mice with a conditional, postnatal KO of GP130 develop emphysema, infection susceptibility because of defective IL-6 signaling, liver and heart damage, and a dysfunctional acute-phase response (Betz et al., 1998). Similarly, loss of function of the downstream signal transducer STAT3 is embryonically lethal in mice (Takeda et al., 1997), whereas hypomorphic STAT3 variants cause immune dysregulation (Steward-Tharp et al., 2014), and conditional osteoblast-specific ablation leads to reduced bone formation (Itoh et al., 2006).

In humans, autosomal-dominant (AD) hyper-IgE syndrome (HIES), caused by dominant-negative *STAT3* mutations, is a complex immunodeficiency that presents with pneumonia, lung abnormalities, high levels of IgE, eosinophilia, eczema, and skeletal and connective tissue abnormalities including retained primary teeth, scoliosis, and craniosynostosis (Smithwick et al., 1978; Höger et al., 1985; Gahr et al., 1987; Grimbacher et al., 1999a, 2005; Holland et al., 2007; Minegishi et al., 2007; Miller et al., 2017). Other syndromes associated with marked IgE elevation and immune deficiency include *DOCK8* deficiency (Engelhardt et al., 2009; Zhang et al., 2009) and *PGM3* deficiency (Sassi et al., 2014; Zhang et al., 2014). Defects in *TYK2* seem to be only rarely associated with HIES (Minegishi et al., 2006; Kreins et al., 2015).

Recently, recessive loss-of-function mutations of *IL11RA*, encoding IL-11RA, were identified in patients with craniosynostosis and dental anomalies (CRSDA; Online Mendelian Inheritance in Man accession no. 614188; Nieminen et al., 2011; Keupp et al., 2013; Papachristoforou et al., 2013; Miller et al., 2017). The clinical features include multi-suture craniosynostosis, maxillary hypoplasia, delayed tooth eruption, supernumerary teeth, and minor digit abnormalities. Mutant mice have increased trabecular bone volume with defective differentiation of osteoclast precursors (Sims et al., 2005), whereas the delayed tooth eruption found in individuals with *IL11RA* mutations is probably caused by reduced bone resorption in the jaw.

Here, we identify a patient with a causative homozygous mutation in *IL6ST*, encoding the human cytokine receptor subunit GP130, presenting with immunodeficiency and skeletal abnormalities including craniosynostosis, and demonstrate associated defects in IL-6, IL-11, IL-27, and OSM signaling.

## Results and discussion

### Patient summary

**Figure 1.**
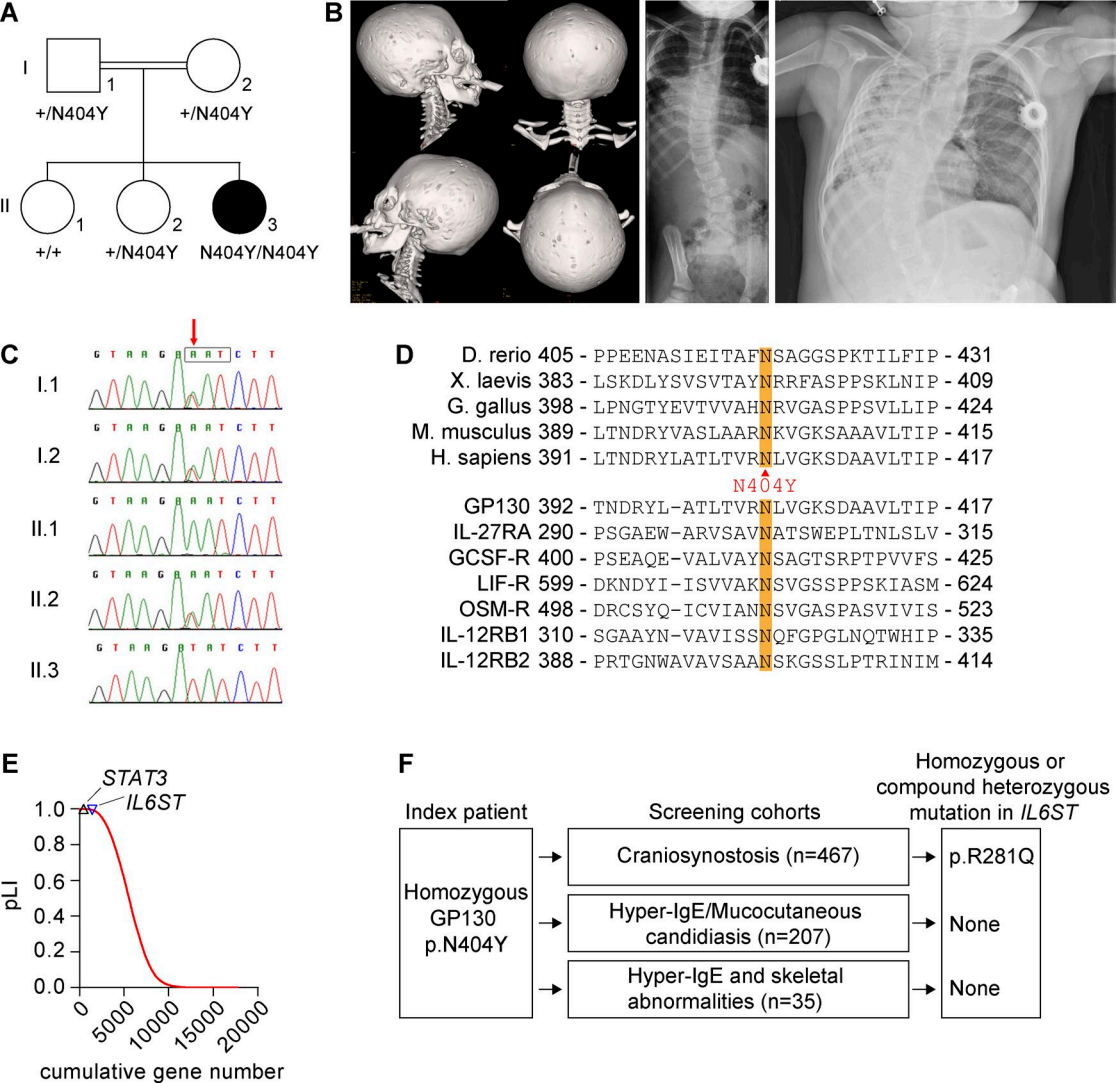
**Genetic analysis of *IL6ST*.** (A) P1 showing pedigree and segregation of *IL6ST* alleles. Roman numerals (I or II) indicate generations, and Arabic numerals designate individuals (1, 2, or 3). Closed symbols identify the affected individual P1. (B, left) 3D computed tomographic reconstruction of skull of P1 showing pansutural synostosis. (Middle) Posteroanterior radiograph at age 2.8 yr demonstrating scoliosis. (Right) Chest radiograph at age 3.9 yr showing pneumonia, bronchiectasis, and scoliosis. (C) Dideoxy sequencing of P1 family, showing segregation of the c.1210A>T variant (red arrow). The box encloses the asparagine codon. (D) Alignment of GP130 protein sequence showing conservation of amino acid p.N404 among species (top) and across the class of tall cytokine receptors (bottom). Substitution to tyrosine (Y) is shown between the panels. (E) pLI of 17,739 human genes based on ExAC data. *IL6ST* and *STAT3* genes are highlighted indicating their strong intolerance to loss-of-function variation. (F) Screening strategy within cohorts of patients with craniosynostosis and/or immunodeficiency (HIES and mucocutaneous candidiasis). A homozygous c.842G>A; p.R281Q substitution identified in the craniosynostosis cohort was classified as a variant of unknown significance and is not further discussed.

The index patient 1 (P1), the female offspring of consanguineous (first cousin) parents of south Asian origin (Fig. 1 A), presented with skeletal abnormalities, including severe craniosynostosis and progressive scoliosis (Fig. 1 B). From the age of 6 mo, she developed multiple early, severe, and complicated recurrent respiratory tract infections, including pneumonia that needed antibiotic treatment, multiple hospitalizations, and intensive care. Then, the patient developed severe bronchiectasis and required oxygen supplementation. At 1 yr of age, eye infections were apparent, and bilateral corneal ulcers were noted. Other severe infections included periorbital cellulitis caused by group A *Streptococci*. She presented with eczema at the age of 2 yr as well as increasingly high IgE levels (maximum >5,000 IU/ml) and intermittent eosinophilia (for immunological characteristics of P1, see Tables 1, 2, and 3). This is reflected by a high NIH HIES score (Grimbacher et al., 1999b) of 40. A detailed patient history is provided in Materials and methods. No other family members were affected.

**Table 1.** Lymphocyte subsets of P1 (p.N404Y)

	1.9 yr of age		5 yr of age		6.1 yr of age	
	Patient	Reference range	Patient	Reference range	Patient	Reference range
Absolute lymphocyte count (G/liter)	7.4	2.7–11.9	4.5	1.7–6.9	3.9	1.1–5.9
CD3^+^ T cells (cells/μl)	4,600	1,400–8,000	2,831	900–4,500	2,876	700–4,200
CD3^+^HLA-DR^+^ (%)			6	3–13	16	3–14
CD4^+^ T cells (cells/μl)	3,030	900–5,500	1,716	500–2,400	1,244	300–2,000
CD45RA^+^CCR7^+^ (%)					78.5	57.4–84.9
CD45RA^−^CCR7^+^ (%)					9.8	11.3–26.7
CD45RA^−^CCR7^−^ (%)					9.1	3.3–15.2
CD45RA^+^CCR7^−^ (%)					1.7	0.4–2.6
CD8^+^ T cells (cells/μl)	1,570	400–2,300	1,009	300–1,600	1,459	300–1,800
CD45RA^+^CCR7^+^ (%)					44.9	28.4–80.6
CD45RA^−^CCR7^+^ (%)					1	1–4.5
CD45RA^−^CCR7^−^ (%)					6.7	6.2–29.3
CD45RA^+^CCR7^−^ (%)					43.1	9.1–49.1
CD4/CD8	1.9	1–3	1.7	1–2.1	0.85	0.9–2.6
CD56^+^CD16^+^ NK cells (cells/μl)	110	61–510	99	70–590	231	70–590
CD19^+^ B cells (cells/μl)	1,560	529–1,930	**1,532**	323–1,000	788	212–796
CD27^−^IgD^+^ naive (%)			**96**	70–86	**93**	63–89
CD27^+^IgD^+^ nonswitched memory (%)			**3**	7–15	3	3–19
CD27^+^IgD^−^ class-switched memory (%)			**1**	4–16	**3**	4–17

Abnormal results are shown in bold.

**Table 2.** Immunoglobulins and serum antibody responses of P1 (p.N404Y)

	1.2 yr of age		1.8 yr of age		5 yr of age		6.1 yr of age	
	Patient	Reference range	Patient	Reference range	Patient	Reference range	Patient	Reference range
IgG (g/liter)	11.5	3.45–12.13	7.81	4.24–10.51	7.8	4.63–12.36	10.45	6.33–12.80
IgA (g/liter)	**1.65**	0.14–1.06	0.84	0.14–1.23	0.25	0.25–1.54	0.31	0.33–2.02
IgM (g/liter)	**1.86**	0.43–1.73	1.23	0.48–1.68	1.07	0.43–1.96	0.85	0.48–2.07
IgE (IU/liter)	43	2–97	**466**	2–97			**>5,000**	2–307
Hib IgG (μg/ml)			6.64	0.16–40.8				
Pneumococcal (titer)								
IgG			1,280	640				
IgG2			40	40				
Tetanus antibody (IU/ml)			0.35	0.04–3.92				

Abnormal results are shown in bold.

**Table 3.** Eosinophils and neutrophil respiratory burst in P1 (p.N404Y)

	1.8 yr of age		6.1 yr of age	
	Patient	Reference range	Patient	Reference range
Eosinophils (G/liter)	**3.36**	0.0–0.8	**2.37**	0.0-0.8
Neutrophil respiratory burst (DHR)	63	>40		

Abnormal results are shown in bold.

### Identification of *IL6ST* mutation and prediction of the mutational impact

Initial genetic investigation for causes of craniosynostosis, including sequencing of fibroblast growth factor receptor 1 (*FGFR1*), *FGFR2*, and *FGFR3*, and array comparative genomic hybridization yielded negative results. Exome sequencing of DNA did not identify known genetic causes of craniosynostosis (Miller et al., 2017). Assuming a recessive mode of inheritance and homozygosity of the causative mutation, the exome data identified 29 regions with homozygous stretches >5 Mb (not depicted). In total, there were 375 homozygous variants (with ≥4 variant reads). After removal of synonymous variants and changes found within in-house exome-sequencing data, 49 nonsynonymous variants remained (Table S1). The top ranking candidate (determined by six predictors of the functional effect of amino acid substitutions; Fu et al., 2013) was a homozygous variant (c.1210A>T) in *IL6ST*, predicting a substitution of tyrosine for asparagine (p.N404Y). Dideoxy sequencing of samples from the family showed that both parents were heterozygotes and that the variant segregated in a homozygous state only to P1 but not to the other two healthy siblings (Fig. 1 C).

The c.1210A>T variant was not present in any database (including National Heart, Lung, and Blood Institute Exome Variant Server, INTERVAL cohort, UK10K consented exomes, 1,000 Genomes, Exome Aggregation Consortium [ExAC] v0.3.1, and gnomAD) covering >120,000 exomes or genomes and different populations. The amino acid N404 in GP130 is evolutionary highly conserved across species as well as across related cytokine receptors such as IL-27RA/WSX1, IL-12RB1, and GCSF-R encoded by *CSF3R* (Fig. 1 D; Xu et al., 2010), suggesting a conserved structural role of this residue. p.N404Y is predicted to be damaging by several scores including SIFT and Polyphen2 (Table S1). No other predicted pathogenic mutations were detected in candidate genes such as *IL6RA*, *STAT3*, *TYK2*, *IL11RA*, or *IL27RA*. A homozygous variant was detected in *DOCK8*. However, it was considered unlikely to be a major contributor to the phenotype (Table S1).

The constraint metric for *IL6ST* based on the ExAC dataset (probability of loss-of-function intolerance [pLI] = 0.995) indicates strong selection against predicted loss-of-function mutations (Fig. 1 E; Lek et al., 2016). No deletions or pathogenic variants in *IL6ST* are annotated in the CLINVAR database. In contrast, *IL6ST* variants predicting GP130 gain of function have been described in several tumors, in particular hepatocellular adenomas (Pilati and Zucman-Rossi, 2015). However, the p.N404Y substitution is absent in the Catalog of Somatic Mutations in Cancer (COSMIC) database.

### Resequencing of *IL6ST* and exome screening

In an effort to identify additional cases, we screened *IL6ST* for homozygous or compound heterozygous variants either by direct resequencing (467 patients with craniosynostosis, mutation negative for the major known causes) or by interrogation of existing exome data (207 patients with HIES or chronic mucocutaneous candidiasis; 35 patients from 25 families with HIES and skeletal abnormalities; summarized in Fig. 1 F). No convincingly pathogenic rare homozygous or compound heterozygous variants were found in these cohorts.

### Differential effects of GP130 variants on IL-6, IL-11, IL-27, OSM, and LIF signaling

**Figure 2.**
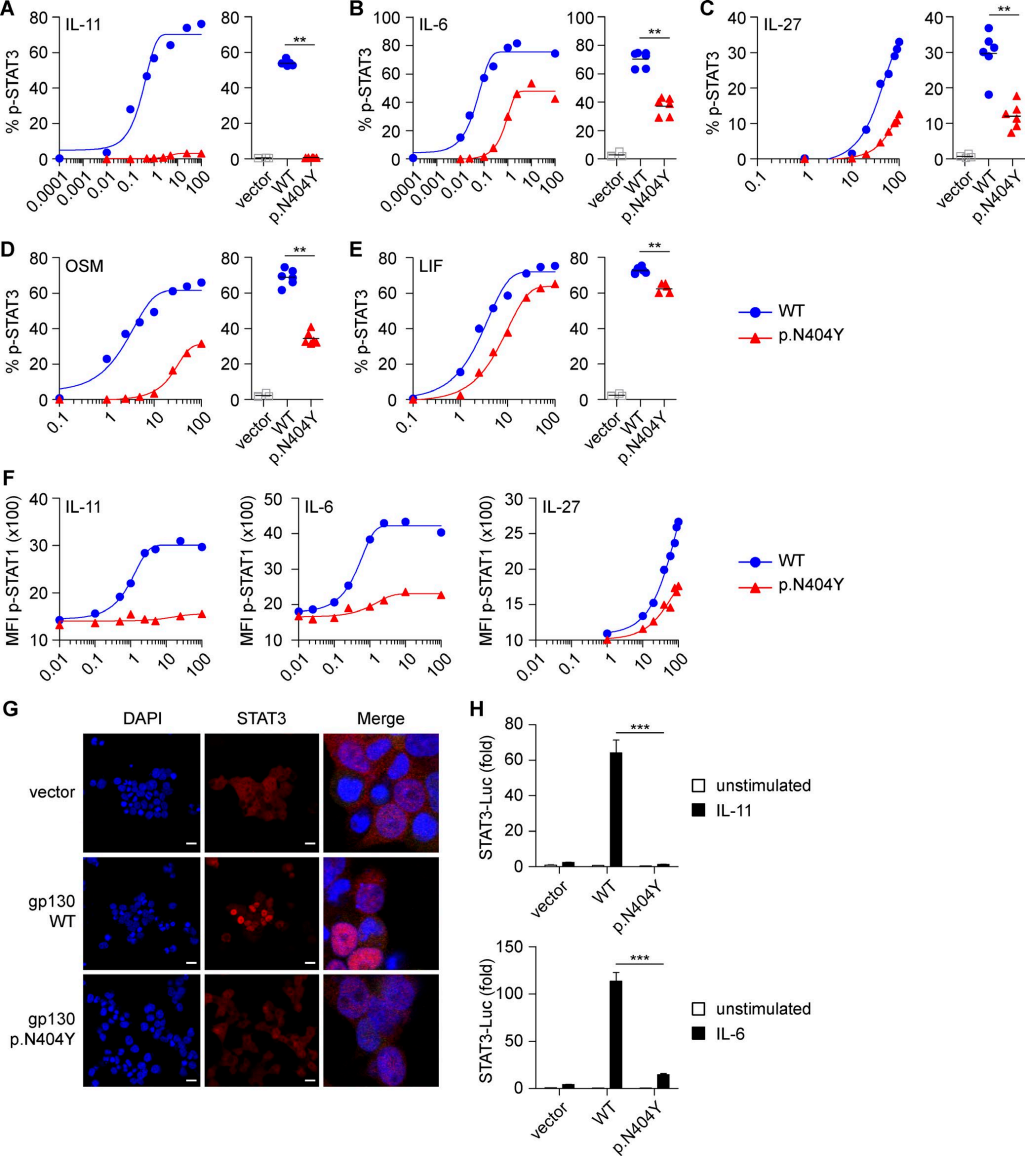
**The GP130 p.N404Y substitution causes defective signaling by IL-11, IL-6, IL-27, OSM, and LIF.** (A–E) HEK293 GP130-KO cells were transfected with empty vector control or plasmids encoding GP130 WT or the patient variant p.N404Y. Cells were stimulated with the indicated concentrations (ng/ml) of IL-11 (A), IL-6 (B), IL-27 (C), OSM (D), or LIF (E) for 15 min and analyzed for STAT3 phosphorylation (p-STAT3) by Phosflow. For assessment of IL-11 and IL-6 signaling, cells were cotransfected with plasmids encoding IL-11RA and IL-6RA, respectively. Co-transfection with GFP allowed gating on successfully transfected cells. Representative titration curves (on the left in each panel) are shown for each ligand and are representative of two independent experiments. Curve fitting is by nonlinear regression. Quantification (on the right in each panel) is based on four to six independent experiments per cytokine at one concentration (IL-11, 1 ng/ml; IL-6, IL-27, OSM, and LIF, all 100 ng/ml). (F) Experiments with HEK293 GP130-KO cells performed as in A–C. Cells were assayed for phospho-STAT1 (p-STAT1). Titration curves are representative of two independent experiments. MFI, mean fluorescence intensity. (G) Immunofluorescence staining of HEK293 GP130-KO cells, plated in chamber slides and transfected as in A. Cells were analyzed for STAT3 nuclear translocation using confocal microscopy. Bars, 50 µm. Images on the right are merged and magnified. Images are representative for three independent experiments. (H) HEK293 cells were cotransfected with luciferase (Luc) reporters, GP130 variants, and IL-11RA or IL-6RA expression vectors, respectively. After 24 h, cells were stimulated with 1 ng/ml IL-11 (top) or 0.5 ng/ml IL-6 (bottom) for 6 h, and induction of STAT3 reporter (relative to constitutively expressed Renilla luciferase) was determined. Results are expressed as fold-induction compared with unstimulated vector control and are pooled data from three independent experiments with three to six technical replicates each. Data represent mean with SEM. Differences were investigated by Mann-Whitney *U* test. **, P < 0.01; ***, P < 0.001.

To compare the in vitro effects of the likely pathogenic p.N404Y variant on signaling of different cytokines that require GP130, we created a GP130-deficient HEK293 cell line (HEK293 GP130-KO) using CRISPR/Cas9 (Fig. S1, A–E). This cell line does not phosphorylate STAT1 or STAT3 in response to stimulation with IL-6 (Fig. S1, D and E), IL-11, IL-27, OSM, or LIF (not depicted) but has normal STAT3 signaling in response to type 1 IFN and normal STAT1 in response to IFN-γ (Fig. S1 E). Transfection with GP130 WT restores GP130-dependent signaling (Fig. 2). We excluded differences between the surface expression of GP130 WT and the variant p.N404Y by flow cytometry, suggesting that p.N404Y does not cause mRNA or protein instability (Fig. S1 F). Titration studies on transfected GP130-KO cells revealed that the variant showed an absent STAT3 phosphorylation in response to IL-11 stimulation (Fig. 2 A) and significantly reduced STAT3 phosphorylation after stimulation with IL-6, IL-27, or OSM (Fig. 2, B–D). Interestingly, the LIF response was largely intact (Fig. 2 E). The p.N404Y variant also exhibited defective IL-11–induced STAT1 phosphorylation and a reduced STAT1 response to IL-6 or IL-27 (Fig. 2 F).

Next, we used confocal microscopy to assess the impact of p.N404Y on IL-6–mediated STAT3 nuclear translocation. In p.N404Y-transfected HEK293 GP130-KO cells, we observed a defective cytoplasmic to nuclear STAT3 translocation in response to IL-6 (Fig. 2 G). Furthermore, we investigated the functional consequences of GP130 p.N404Y in a STAT3 luciferase reporter assay. The p.N404Y variant caused defective luciferase induction after IL-6 and IL-11 stimulation (Fig. 2 H).

We validated the defective signaling of the p.N404Y variant in response to IL-11 by Western blotting (not depicted). Pulldown experiments suggested that the signaling defect is not caused by defective heterodimerization of the GP130 p.N404Y variant with IL-6RA or IL-11RA (not depicted).

### Defects in the IL-6 response in primary immune cells of GP130 p.N404Y

**Figure 3.**
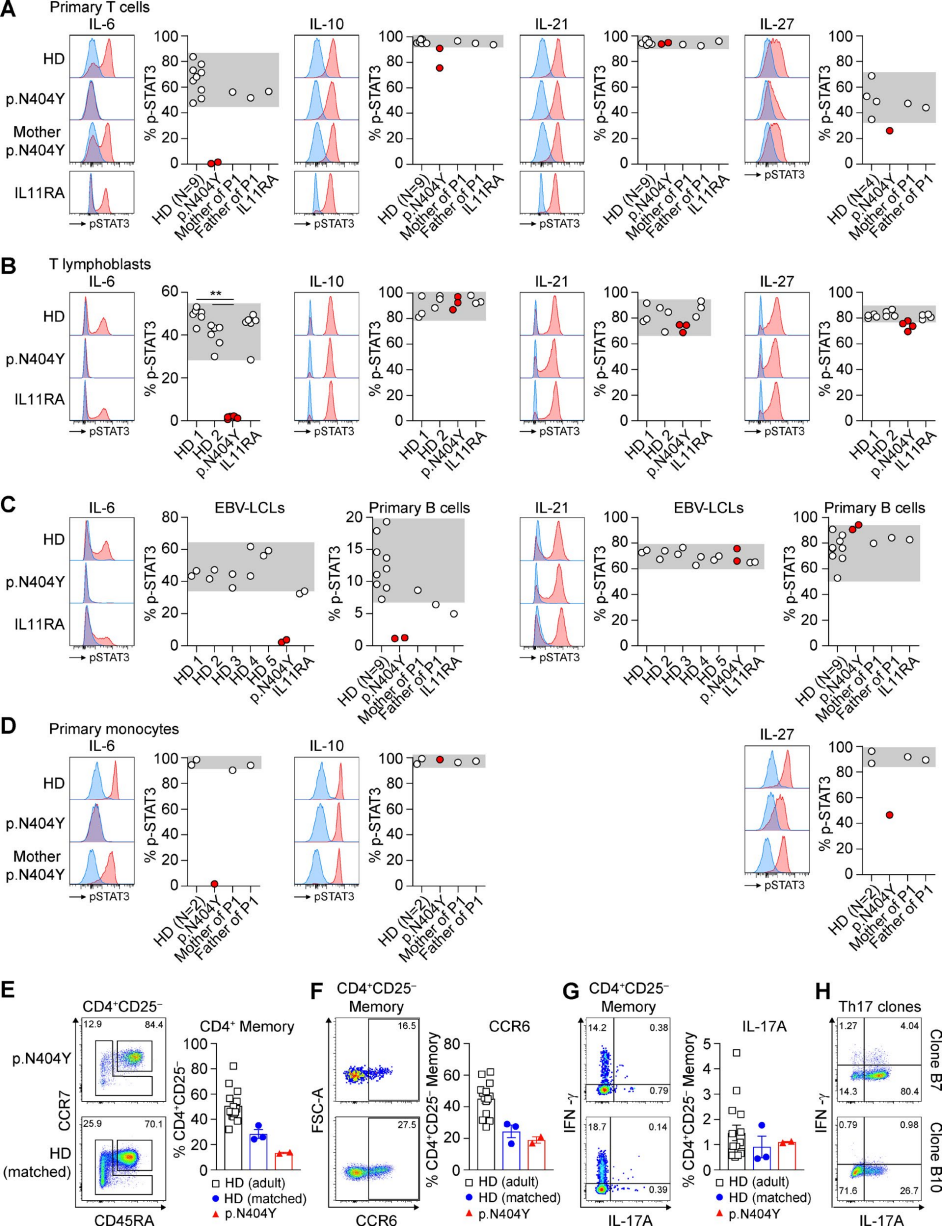
**The GP130 p.N404Y substitution abrogates IL-6 signaling in primary T cells, B cells, and monocytes.** (A–D) Primary immune cells were stimulated for 15 min with 100 ng/ml IL-6, 50 ng/ml IL-10, 100 ng/ml IL-21, or 100 ng/ml IL-27, and STAT3 phosphorylation (p-STAT3) was analyzed using intracellular staining and flow cytometry. Representative histograms are shown (blue, unstimulated; red, cytokine stimulated). Results show percent phospho-STAT3^+^ cells based on the unstimulated condition. Gray areas indicate range found in healthy individuals. (A) Levels of STAT3 phosphorylation were assessed in T cells (CD3^+^/CD19^−^ lymphocyte gate) in a whole-blood assay. Analysis was performed on nine healthy donors (HD), patient P1 (p.N404Y), the parents of P1, and a patient with a homozygous IL-11RA substitution (p.D297Y). Blood samples of P1 were independently analyzed on two occasions, separated by 13 mo. (B) Comparison of phospho-STAT3 levels in T lymphoblasts of two healthy donors (age matched to P1), P1, and the IL-11RA patient, which were generated from frozen PBMCs by PHA/IL-2 expansion. Data are a pooled summary result from three independent experiments with one, two, and four replicates each. Differences were investigated by Mann-Whitney *U* test. **, P < 0.01. (C) Assessment of LCLs generated from five healthy donors, P1, and the IL-11RA patient. Experiments with LCLs were performed twice, independently. Analysis of primary B cells was performed in the whole-blood assay described in A. (D) STAT3 phosphorylation in primary monocytes based on the whole-blood assay described in A. (E–G) Freshly isolated PBMCs were surface stained using fluorophore-conjugated antibodies and analyzed by flow cytometry. HD (adult), *n* = 15; HD (age matched), *n* = 3; P1 (p.N404Y), mean of two technical replicates of two independent measurements. Representative dot blots of P1 (p.N404Y; top) and the age-matched healthy donor (bottom) are shown. Bar graphs summarize measurements from several individuals. (E) CD45RA and CCR7 surface expression in CD3^+^CD4^+^CD8^−^CD25^−^ T cells showing the percentage of naive (CD45RA^+^CCR7^+^) and memory (CD45RA^−^CCR7^+/−^ central memory/effector memory including CD45RA^+^CCR7^−^ TEMRA) cells. (F) Dot plot presentation and bar graph showing the expression of CCR6 in CD4^+^CD25^−^ memory T cells as assessed by surface staining. FSC, forward scatter. (G) Intracellular cytokine staining for IFN-γ and IL-17A after PMA/ionomycin stimulation of freshly isolated PBMCs showing frequencies of CD4^+^CD25^−^ memory T cells. (H) Dot plot presentation showing intracellular IFN-γ and IL-17A after PMA/ionomycin stimulation of two IL-17A–producing single-cell Th cell clones prepared from the Th17 cell–enriched memory compartment, analyzed 28 d after isolation and single-cell cloning. Error bars represent mean ± SEM.

To investigate the functional consequences of the GP130 p.N404Y substitution in primary immune cells, we stimulated whole blood of P1 as well as controls with the GP130-dependent cytokines IL-6 and IL-27 for 15 min and measured STAT3 phosphorylation using intracellular staining and flow cytometry (Fig. 3). P1 displayed a complete loss of IL-6 signaling in primary T cells, B cells, and monocytes (Fig. 3, A, C, and D). Immune cells from healthy donors or a patient with a previously unpublished homozygous c.889G>T (p.D297Y; see the Case studies section of Material and methods for phenotype) mutation in *IL11RA* responded to IL-6 stimulation. We confirmed the defective IL-6 signaling in P1-derived T lymphoblasts (Fig. 3 B) as well as EBV-transformed lymphoblastoid cell lines (LCLs; Fig. 3 C). Cellular responses to IL-27 were impaired in P1 (particularly in monocytes) but not abolished (Fig. 3 D). Stimulation with IL-10 and IL-21, as controls for the induction of phospho-STAT3, showed a normal response in P1 and controls.

### Immunodeficiency caused by the p.N404Y substitution

To understand the immune defect in P1, we characterized leukocyte and lymphocyte subsets. The absolute numbers of T, B, and NK lymphocytes of P1 in the peripheral blood were largely normal, but a reduced fraction of class-switched memory B cells were noted (Table 1). The reduced proportion of memory B cells in P1 (Table 1) is reminiscent of patients with *STAT3*-HIES and patients treated with anti–IL-6R mAb tocilizumab (Roll et al., 2011) but is not seen in patients with atopic dermatitis and high IgE (Speckmann et al., 2008; Meyer-Bahlburg et al., 2012). This confirms the role of IL-6 signaling for B cell memory. As an indication of a potentially distinct differential B cell defect, in *STAT3*-HIES, poor pneumococcal antibody responses were described requiring intravenous immunoglobulin in 4 of 10 patients (Flinn et al., 2016), whereas in the p.N404Y GP130 patient, a normal pneumococcal response was noted (Table 2).

P1 presented with eosinophilia (maximum 3.36 Gpt/liter) and high IgE (>5,000 IU/ml at 6.1 yr of age), whereas levels of IgM, IgG, and IgA were not affected, and levels of antibody to Haemophilus, Pneumococcus, and Tetanus were each within the normal range (Tables 2 and 3). Given the previously reported role of STAT3 signaling in generating IL-17–producing lymphocytes (de Beaucoudrey et al., 2008; Ma et al., 2008; Milner et al., 2008), we investigated the frequency of T helper type 17 cells (Th17 cells) ex vivo. Within the compartment of CD4^+^CD45RA^−^CD25^−^ memory T cells, we detected CCR6^+^, RORγt-expressing (not depicted), and IL-17A–producing cells (Fig. 3, E–G) and demonstrated the presence of in vivo–differentiated memory Th17 cells in P1 through the isolation of IL-17A–producing single-cell clones (Fig. 3 H). Together, these results are consistent with the absence of fungal infections in P1.

### Delayed or absent acute-phase response in P1

**Figure 4.**
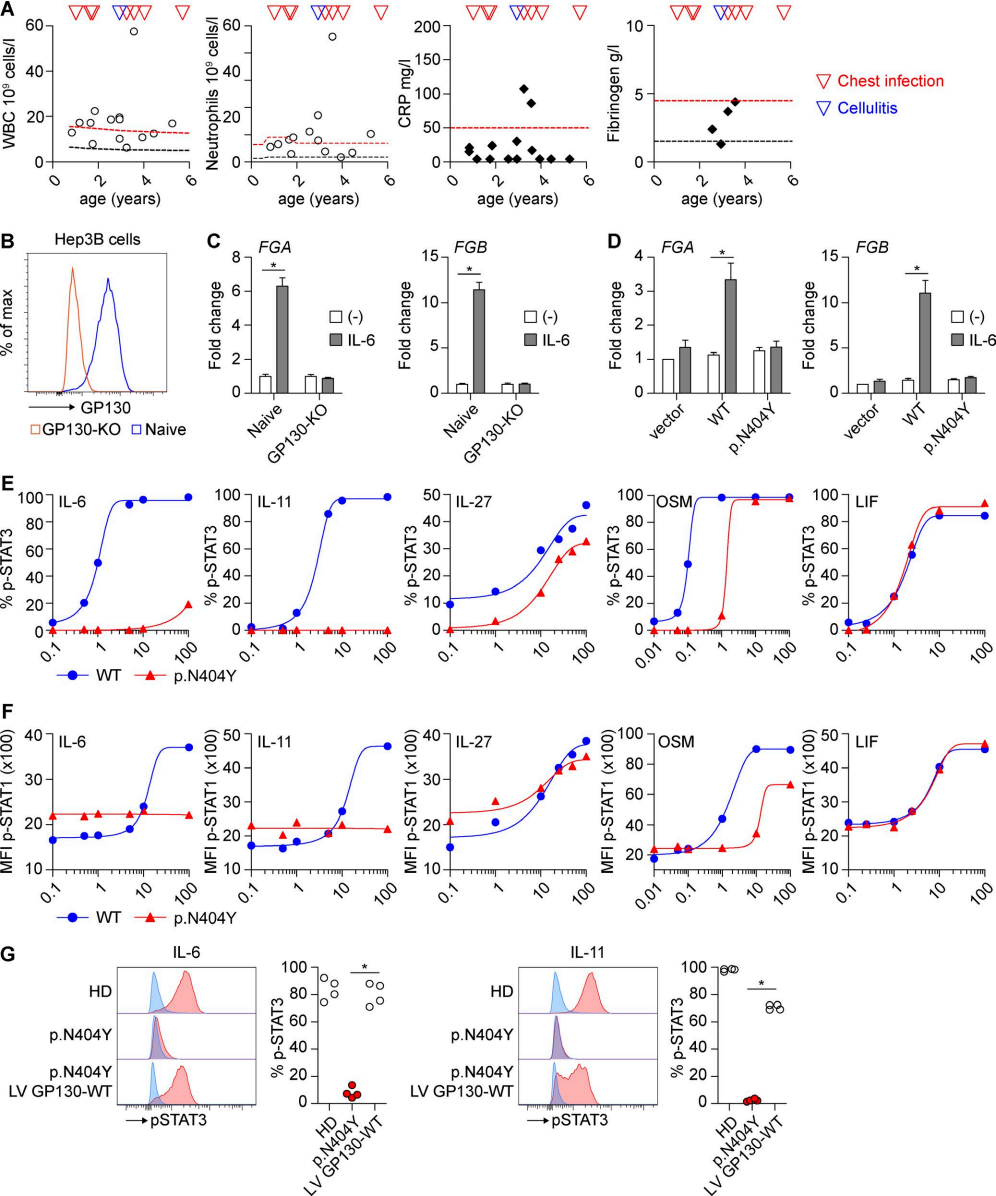
**The GP130 p.N404Y variant causes a defective acute phase response, and P1 primary fibroblasts can be rescued by lentiviral transduction of WT GP130.** (A) Cellular and biochemical response to recurrent chest infections and cellulitis in patient GP130 p.N404Y. White blood cell count (WBC), neutrophils, CRP, and fibrinogen measurements are shown at different time points. Black/red lines indicate 3rd/97th percentile or lower/upper limit of the normal range, respectively. (B) CRISPR/Cas9-mediated KO of GP130 in human hepatoma Hep3B cells results in absent GP130 surface expression. (C) IL-6–mediated production of fibrinogen is GP130 dependent. Hep3B GP130-KO cells were stimulated with IL-6 for 24 h, and expression of the *fibrinogen α chain* (*FGA*; left) and *fibrinogen β chain* (*FGB*; right) was determined by quantitative PCR. Gene expression was determined relative to the housekeeping gene *RPLP0* and expressed as fold-induction compared with unstimulated cells. Data represent summary results from four independent experiments (mean ± SEM). (D) Hep3B GP130-KO cells were transiently transfected with GP130 WT, GP130 p.N404Y-mutant plasmid, and *FGA* (left) and *FGB* (right) gene expression was analyzed after 24 h of IL-6 stimulation. Gene expression was determined relative to the housekeeping gene *RPLP0* and expressed as fold-induction compared with the unstimulated vector control. Data represent pooled results from four independent experiments (mean ± SEM). (E) Fibroblasts of a healthy donor and P1 were stimulated with the indicated concentrations (ng/ml) of IL-6, IL-11, IL-27, OSM, and LIF. The levels of phospho-STAT3 (p-STAT3) were determined after 15-min stimulation by Phosflow. Titration curves are representative of two independent experiments. Curves are fitted by nonlinear/linear regression analysis. (F) Healthy donor and patient GP130 p.N404Y fibroblasts were treated as in E, and STAT1 phosphorylation (p-STAT1) was analyzed using flow cytometry. Titration curves are representative of two independent experiements. MFI, mean fluorescence intensity. (G) Lentiviral transduction of GP130 WT reconstitutes STAT3 phosphorylation in primary fibroblasts with p.N404Y variant. Fibroblasts were stimulated with 30 ng/ml IL-6 and 50 ng/ml IL-11 for 15 min. Quantification is based on four independent experiments. HD, healthy donor; LV, lentiviral. Differences were determined by Mann-Whitney *U* test. *, P < 0.05.

We noted that, despite severe bacterial infections with *Staphylococcus aureus* (culture from cornea and lung), *Streptococcus* group A, or *Streptococcus milleri* that required intensive care and antibiotic treatment and led to repeated leukocytosis and neutrophilia, there was only a delayed and reduced acute phase response. Even with exceptionally high leukemic-like counts (peripheral blood white blood cells, 57 Gpt/liter; and neutrophil count, 46 Gpt/liter), only a moderate and delayed C-reactive protein (CRP) increase and no fibrinogen elevation were found in P1 (Fig. 4 A). Given that IL-6 is the major stimulus for the production of most acute-phase proteins (Gabay and Kushner, 1999), we surmised that the p.N404Y mutation impairs the hepatocyte response. To test this hypothesis in an in vitro model, we created an *IL6ST*/GP130 KO using CRISPR/Cas9 in the human hepatoma cell line Hep3B and confirmed that these cells are defective in the IL-6–mediated induction of fibrinogen (Fig. 4, B and C). GP130-KO cells transfected with GP130-p.N404Y were unable to up-regulate fibrinogen after stimulation with IL-6, whereas transfection of GP130-WT restored the response (Fig. 4 D). This finding suggests that a delayed or absent acute-phase response as a critical innate immune reaction toward bacterial infection contributes to the complex immunodeficiency in P1. Acute-phase proteins such as CRP and fibrinogen bind to the bacterial surface and support opsonization, bacterial uptake, and killing (Gabay and Kushner, 1999). Mice deficient in fibrinogen are more susceptible to group A streptococcal infections, and fibrinogen has direct antimicrobial activity (Påhlman et al., 2013). Early antibiotic therapy for ongoing infections is therefore required in P1, irrespective of normal, potentially false-negative CRP values, to avoid repeat bacterial lung infections causing secondary lung structural damage. Interestingly, a blockade of IL-6 signaling by therapeutic antibodies (Nguyen et al., 2013) or autoantibodies (Puel et al., 2008) was associated with severe bacterial infections and compromised CRP up-regulation, suggesting a shared clinical outcome of gram+ infection susceptibility in IL-6 signaling defects.

### The p.N404Y substitution causes stromal cell dysfunction and can be rescued by WT GP130

Stromal cells express high levels of GP130 and respond to multiple cytokines. Fibroblasts of P1 showed absent or substantially reduced levels of phospho-STAT3 and phospho-STAT1 after IL-11 and IL-6 treatment, moderate reduction after IL-27 and OSM stimulation, and a normal response pattern after LIF treatment (Fig. 4, E and F). Thus, our previous in vitro transfection assay with HEK293 GP130-KO cells (Fig. 2) reflects the response in patient-derived primary fibroblasts.

To confirm the causal relationship between the mutation of GP130 p.N404Y and the defective cytokine response, we expressed WT GP130 in P1 fibroblasts by lentiviral transduction. This rescued the impaired IL-6– or IL-11–mediated STAT3 phosphorylation (Fig. 4 G).

### Structural impact of the mutations on binding of cytokine receptors

Next, we wanted to understand the structural mechanisms by which p.N404Y causes a differential defect in the signaling of cytokines via GP130. Therefore, we investigated the published protein structures of the IL-6/IL6-RA/GP130 hexamer complex (Fig. S2 A; Boulanger et al., 2003; Xu et al., 2010). The N404 residue in GP130 is not exposed at the surface but forms part of a β-turn that comprises N404, L405, V406, and G407 (Fig. S2 B). The amide group of N404 forms a network of hydrogen bonds, mainly with the backbone oxygens of A359, N360, and G361 as well as with the backbone nitrogen atoms of V406 and G407 (Fig. S2 B). As these interactions stabilize the β-turn, the p.N404Y exchange is likely to weaken the secondary structure by disruption of several hydrogen bonds to neighboring backbone atoms. Thus, as supported by our data, the pathogenicity of p.N404 might not be driven by aberrant interaction of specific receptor–ligand pairs but rather by defective secondary and tertiary structure of the receptor.

Our data collectively show that a loss-of-function mutation of *IL6ST*, encoding GP130 p.N404Y, results in a severe syndromic immunodeficiency with skeletal abnormalities and a pleiotropic defect in signaling by multiple cytokines (including IL-6, IL-11, IL-27, and OSM), features that largely phenocopy AD-HIES because of loss-of-function *STAT3* mutations (Holland et al., 2007; Minegishi et al., 2007). The partially overlapping features between the GP130 p.N404Y patient and AD-HIES caused by *STAT3* mutations inform and differentiate aspects of defective cytokine signaling. Aside from diminished IL-6 signaling, defects in IL-10, IL-21, and IL-23 signaling were suggested to explain aspects of HIES (Minegishi and Saito, 2011). For example, increased serum IgE and disturbed B cell differentiation were attributed to abnormal IL-21 signaling in *STAT3*-HIES, and IL-10 signaling defects were associated with its immune dysregulation (Minegishi and Saito, 2011). Our findings now restrict those candidate pathways and suggest that many key features of HIES are likely caused by IL-6 and IL-11 signaling defects. Similar to *STAT3*-HIES (Flinn et al., 2016; Miller et al., 2017), the early lung defects such as bronchiectasis in P1 may be caused by recurrent infections, but additional features, such as lung epithelial protective regeneration or increased matrix metalloprotease activity, as well as scoliosis, might contribute to the extreme phenotype. Staphyloccocal colonization has been noted in keratitis lesions as well as in the lung of P1, but no candidiasis was noted. Reduced Th17 responses are a hallmark of *STAT3*-HIES, causing impaired immune responses to Staphylococci with abscesses in skin and lungs, as well as chronic mucocutaneous candidiasis (de Beaucoudrey et al., 2008; Ma et al., 2008; Milner et al., 2008). The presence of Th17 cells in P1, but defective T cell differentiation in AD-HIES and in human *IL21R* defects, hints at the differential contribution of the IL-21–STAT3 axis (Kotlarz et al., 2014; Ma et al., 2016).

Craniosynostosis and dental abnormalities are a shared phenotype of P1, CRSDA patients associated with *IL11RA* mutations (Nieminen et al., 2011; Keupp et al., 2013; Papachristoforou et al., 2013; Twigg and Wilkie, 2015), and AD-HIES caused by *STAT3* mutations (Miller et al., 2017) but have not been observed in recessive *DOCK8* cases (Engelhardt et al., 2015). This strongly points to disturbance in a shared IL-11/IL-11RA/GP130/STAT3–driven process mediating these phenotypes. In contrast, scoliosis, a skeletal abnormality present in P1 (p.N404Y), is typically associated with *STAT3*-HIES (Grimbacher et al., 1999a) but not with *IL11RA* mutations (Nieminen et al., 2011; Keupp et al., 2013; Papachristoforou et al., 2013). Interestingly, common variants in *IL6* have been linked to scoliosis in genome-wide association studies (Aulisa et al., 2007; Nikolova et al., 2015). This suggests that abnormal IL-11 signaling does not contribute to scoliosis and raises the question of whether scoliosis is a primary connective tissue phenotype of variants in IL-6 signaling itself or a consequence of lung pathology found in HIES caused by STAT3 or GP130 defects. Dysplasia of long bones and the spine are also associated with rare Stüve-Wiedemann syndrome caused by loss-of-function mutations in *LIFR* (Dagoneau et al., 2004; Jung et al., 2010; Mikelonis et al., 2014). The preserved LIF signaling in P1 explains different clinical manifestations of P1 and patients with Stüve-Wiedemann syndrome.

Not surprisingly, *IL6ST* was predicted to be one of the 3,110 as yet undiscovered primary immunodeficiency genes (Itan and Casanova, 2015). The negative outcome of our extensive search across several cohorts with *STAT3*/*DOCK8*/*PGM3* mutation-negative HIES, covering a combined total of over 200 individuals, suggests that immunodeficiency caused by genetic defects in GP130 is very rare. Indeed, *IL6ST* has been screened previously as a candidate gene for both craniosynostosis (Keupp et al., 2013) and primary immunodeficiency (Woellner, 2013) without success. This might be explained by the essential role of GP130 in signaling pathways such as LIF (or CNTF/CT1/CLC) signaling. The high evolutionary constraint pLI score for *IL6ST* (Fig. 1 E) indicates that there is strong negative selection against heterozygous loss-of-function (nonsense or frameshift) mutations. We propose that only particular nonsynonymous variants in GP130 are viable in the homozygous state, depending on the specificity with which they abrogate signaling by different cytokines. In line with this hypothesis, the p.N404Y substitution is defective in IL-6, IL-11, IL-27, or OSM signaling but confers largely normal LIF signaling. This hypothesis is supported by the known activity of LIF, CNTF, CT1, and OSM during the early stages of embryonic development (Kristensen et al., 2005) and the lethal effects of *Lifr* KO in mice (Ware et al., 1995).

## Materials and methods

### Case studies

P1 was born at term after normal pregnancy with congenital left hip dislocation. Her birthweight was 2.78 kg (15.1 percentile according to World Health Organization growth charts). Her perinatal period was otherwise uneventful, and she received standard vaccinations including Bacillus Calmette–Guérin without adverse events. P1 is now 7 yr old.

#### Skeletal system

 An abnormal head shape was noticed by her parents from the age of 6 mo. She was diagnosed at 18 mo with pansutural craniosynostosis, which required surgery at the ages of 2 and 3 yr. Other skeletal malformations included congenital contractures of her right elbow, limited finger extension, and progressive severe scoliosis. Because of congenital left hip dislocation, her left lower limb is shortened by 3 cm.

#### Pulmonary and nonpulmonary systemic infections

Ophthalmic infections were reported at the initial and subsequent presentations. At the age of 6 mo, P1 started to have recurrent chest infections and subsequently developed bronchiectasis of her right lung. She had to be hospitalized on several occasions. At the age of 1.5 yr, a transient low-level EBV viremia was noted.

At the age of 3.5 yr, she was admitted to the hospital with sepsis, right tension pneumothorax, and right-sided empyema requiring intravenous antibiotics, ventilation, chest drain, and intensive care. *S. milleri* was isolated from the pleural fluid culture. After multiple infections, her chest radiograph showed progressive opacification of her right lung. Consistent with a reduced pulmonary functional capacity, she requires oxygen supplementation (0.8 liter/min) at night. Bronchoscopy showed normal airway anatomy, excluding a congenital tracheal or bronchial abnormality as an alternative noninfective explanation for the progressive pulmonary changes. She started azithromycin as prophylaxis for her chest infections at the age of 4.5 yr, with a good response.

After the second cranial surgery, P1 developed orbital cellulitis and severe sepsis, caused by a group A streptococcal infection that spread into the central nervous system leading to secondary superior sagittal sinus thrombosis and left-sided middle cerebral artery territory venous infarct associated with a right-sided hemiplegia. During all reported infectious episodes, P1 never developed a fever.

#### Skin infections

From the age of 2 yr, P1 suffered from eczema with extensive excoriated lesions and secondary skin infections. She is on regular antihistamines and topical treatment with acceptable control.

#### Neurological

P1 has mild to moderate cognitive and motor delay. She started to walk at the age of 2 yr and spoke a few three-word phrases at the age of 5.5 yr. Secondary to the middle cerebral artery territory infarct, she suffers from spasticity of the right upper limb and increased tone in the right lower leg. There is no history of cramps.

#### Case study IL-11RA p.D297Y

The patient was diagnosed with CRSDA caused by a previously unpublished homozygous c.889G>T (p.D297Y) mutation in *IL11RA*. Because of multi-suture craniosynostosis, he required posterior vault expansion for raised intracranial pressure at 5 yr of age and underwent monobloc midfacial advancement. The patient is now 15 yr old.

### Clinical studies

The clinical studies were approved by Oxfordshire Research Ethics Committee B (reference C02.143) and the London Riverside Research Ethics Committee (reference 09/H0706/20). Subjects were enrolled into the craniosynostosis cohort based on referral from a craniofacial unit or clinical genetics department, with craniosynostosis proven on either plain radiographs or computed tomography of the skull.

DNA was extracted from venous blood collected into EDTA, LCLs, or cultured fibroblasts established from skin biopsies. All DNA was extracted using the Nucleon Blood and Cell Culture DNA extraction kit (Gen-Probe Inc.) according to the manufacturer’s instructions.

Healthy volunteer donors were recruited as part of the Oxford Gastrointestinal Illness Biobank (REC 11/YH/0020) or obtained as leukocyte cones from the UK blood donor bank. Informed consent for participation in this study was obtained from healthy donors, patients, or their parents.

### Exome and genome sequencing

3 µg of genomic DNA from P1 was captured using a TruSeq kit (v.2; Illumina) and sequenced on a HiSeq2000 platform (Illumina). Reads were mapped with Novoalign (Novocraft Technologies), and variants were called using SAMtools (v0.1.19) and annotated using ANNOVAR (Wang et al., 2010). Homozygous variants (called if variant reads accounted for >80% of total reads; total 477) were prioritized by restricting analysis to changes with greater than four variant read calls, in coding regions only, that were predicted to change the amino acid sequence (total of 49 variants; Table S1). The cDNA numbering for the *IL6ST* variant identified is based on transcript NCBI RefSeq accession no. NM_002184/Ensembl accession no. ENST00000381298.6, beginning at the ATG start codon. Exome- and genome-sequencing data from 35 patients from 25 families with HIES phenotype were screened for mutations in *IL6ST*. These individuals had been referred for HIES with atopy, elevated serum IgE, and connective-tissue or skeletal abnormalities and were *STAT3* WT.

### Targeted and Sanger sequencing

Resequencing of *IL6ST* (covering all 18 exons) in 467 subjects with craniosynostosis (negative for mutations in the major causative genes) was performed using either the Access Array integrated fluidic circuit system (Fluidigm) or molecular inversion probes (O’Roak et al., 2012) followed by analysis on the Ion PGM system (Life Technologies Ltd). Sanger sequencing was performed using standard protocols. Primer sequences and PCR amplification conditions are available on request. Immunodeficiency patients (HIES and mucocutaneous candidiasis; 207 subjects) were screened using HIES/chronic mucocutaneous candidiasis panels based on HaloPlex (Agilent) and MiSeq (Illumina) technologies.

### Cell purification and culture

Hep3B cells were obtained from the European Collection of Authenticated Cell Cultures and maintained in DMEM (Sigma-Aldrich) supplemented with 10% FCS. HEK293 were cultured in DMEM supplemented with 10% FCS. Primary human skin fibroblasts from a healthy individual (GM05659) were obtained from Coriell Cell Repositories and cultured in DMEM with 10% FCS. Primary fibroblasts from P1 were generated from a skin biopsy. PBMCs were isolated by density gradient centrifugation (Lymphoprep; StemCell Technologies, Inc.) and cultured in RPMI1640 medium with glutamine (Sigma-Aldrich) supplemented with 10% FCS. LCLs from patients and five healthy donors were generated from PBMCs with supernatant from the EBV-producing marmoset cell line B95-8 according to standard protocols. LCLs were cultured in RPMI medium with 10% FCS. Th cell subsets were FACS sorted according to the following surface marker expression: CD3^+^CD4^+^CD8^−^CD25^−^CD45RA^−^CCR7^+/−^ including CD45RA^+^CCR7^−^ effector memory CD45RA-expressing T cells TEMRA (total memory T cells) and CD3^+^CD4^+^CD8^−^CD25^−^CD45RA^−^CCR7^+/−^CXCR3^−^CCR6^+^CCR4^+^ including CD45RA^+^CCR7^−^ TEMRA (enriched in Th17 cells). Cell sorting was performed using a FACSAria III flow cytometer (BD Biosciences). Flow cytometry data were analyzed with FlowJo software (Tree Star). T lymphoblasts were generated from FACS-sorted CD4^+^ memory T cells (CD3^+^CD4^+^CD25^−^CD45RA^−^CCR7^+/−^ including CD45RA^+^CCR7^−^ TEMRA) and expanded with 1 µg/ml PHA (Gibco), 500 U/ml IL-2 (supernatant of the IL2T6 line), and irradiated allogenic feeder cells. T cells were cultured in RPMI1640 medium with glutamine (Sigma-Aldrich) supplemented with 5% human serum (NHS Blood Center Oxford), 1% sodium-pyruvate (Gibco), 1% nonessential amino acids (Gibco), 1% penicillin/streptomycin (Gibco), and 50 µM β-mercaptoethanol (Sigma-Aldrich). The following antibodies for FACS sorting were used: anti-CD3–PE/Dazzle 594 (clone UCHT1; Biolegend), anti-CD4–BV510 (clone OKT4; Biolegend), anti-CD8–AF700 (clone SK1; Biolegend), anti-CD25–AF647 (clone M-A251; Biolegend), anti-CD45RA–BV650 (clone HI100; Biolegend), anti-CCR7–BV421 (clone G043H7; Biolegend), anti-CD14–APC (clone 18D11; ImmunoTools), anti-CD19–APC (clone LT19; ImmunoTools), and anti-CD56–APC (clone MEM-188; ImmunoTools).

### Ex vivo T cell phenotyping

The expression of surface markers was analyzed by staining for 15 min at room temperature in PBS supplemented with 0.5% (vol/vol) human serum. For the exclusion of dead cells during the analysis, cells were stained before fixation using Fixable Viability Dye eFluor 780 (eBioscience) according to the manufacturer’s instructions. The following fluorophore-conjugated antibodies were used for analysis: anti-CD3 (clone UCHT1; BD Biosciences), anti-CD4 (clone RPA-T4; Biolegend), anti-CD8 (clone SK1; Biolegend), anti-CD14 (clone M5E2; Biolegend), anti-CD19 (clone SJ25C1; BD Biosciences), anti-CD25 (clone MA-A251; Biolegend), anti-CD45RA (clone HI100; Biolegend), anti-CD56 (clone NCAM16.2; BD Biosciences), anti-CD127 (clone A019D5; Biolegend), anti-CCR4 (clone 1G1; BD Biosciences), anti-CCR6 (clone G034E3; Biolegend), anti-CCR7 (clone G043H7; Biolegend), anti-CCR9 (clone L053E8; Biolegend), anti-CCR10 (clone 314305; R&D), anti-CXCR3 (clone 1C6; BD Biosciences), anti-CXCR5 (clone J252D4; Biolegend), and anti-CRTh2 (clone BM16; BD Biosciences).

For intracellular cytokine staining, cells were stimulated for 5 h with 0.2 µM PMA (cat. no. P8139; Sigma-Aldrich) and 1 µg/ml ionomycin (cat. no. I0634; Sigma-Aldrich) in the presence of 10 µg/ml brefeldin A (cat. no. B7651; Sigma-Aldrich) for the final 2.5 h of culture. Cells were fixed and permeabilized with Cytofix/Cytoperm (BD Biosciences) according to the manufacturer’s instructions. For the exclusion of dead cells during the analysis, cells were stained before fixation using Fixable Viability Dye eFluor 780 according to the manufacturer’s instructions. The following fluorophore-conjugated anti-cytokine antibodies were used for analysis: anti–IL-4 (clone MP4-25D2; BD Biosciences), anti–IL-10 (clone JES3-9D7; eBioscience), anti–IL-13 (clone 85BRD; eBioscience), anti–IL-17A (clone eBio64DEC17; eBioscience), anti–IFN-γ (4S.B3; Biolegend), and anti-TNF (clone MAb11; Biolegend). Flow cytometry data were analyzed with FlowJo software.

### T cell single-cell cloning

Single T cell clones were generated by limiting dilution in 384-well plates (Corning) using 1 µg/ml PHA (Gibco) and 2.5 × 10^4^ irradiated allogeneic (45 Gy) PBMCs per well. After 14 d of culture, single-cell clones were transferred to 96-well U-bottom plates and further expanded for a total of 21–28 d in IL-2–containing medium (500 U/ml; supernatant of IL2T6 line). The IL2T6 cell line was provided by F. Sallusto (Università della Svizzera italiana, Bellinzona, Switzerland; and ETH Zurich, Zurich, Switzerland).

### Cytokine stimulation

Cells were stimulated with the indicated concentrations (ng/ml) of recombinant human IL-6, IL-21, IL-22, IL-27, OSM, LIF, IFN-γ, FGF-basic, and epidermal growth factor (all Peprotech) and IL-10, IL-11, and universal type I IFN Protein (all R&D).

### Generation of HEK293 and Hep3B GP130-KO cell lines

HEK293 and Hep3B GP130 KO cell lines were generated using CRISPR/Cas9 following published protocols (Ran et al., 2013). Guide RNA sequences were designed with the CRISPR Design tool (Massachusetts Institute of Technology) and cloned into pSpCas9(BB)-2A-GFP (PX458), which was a gift from F. Zhang (Massachusetts Institute of Technology, Cambridge, MA; plasmid no. 48138; Addgene). Guide RNA clones were verified by sequencing (target sequences: 5′-GGACCAAAGATGCCTCAACT-3′ or 5′-GTTTAGGATTCGCTGTATGA-3′). The resulting plasmid was transfected with Lipofectamine 2000 in HEK293 and Hep3B cells. Cells were expanded and subsequently FACS sorted for the GP130-absent cell population. FACS-sorted Hep3B cells were subjected to single-cell cloning by limiting dilution and plated in 384-well plates together with irradiated feeder cells from a naive Hep3B cell line. Clones were screened for absence of GP130 surface expression using flow cytometry. Dideoxy sequencing of FACS-sorted/cloned cells confirmed disruption of GP130 coding sequences (Fig. S1 C). Staining of surface GP130 was performed with anti-GP130–biotin (AN G30; eBioscience) followed by staining with appropriate streptavidin-conjugated fluorochromes.

### Plasmid and lentivirus generation

Sequences encoding WT or mutant GP130, IL-6RA, or IL-11RA were cloned into the pcDNA3.1(+) vector without C- or N-terminal tags (GenScript). Sequences were verified by Sanger sequencing after each transformation step. The WT *IL6ST* gene was custom cloned into pLENTI7.3V5-DEST (Geneart; Thermo Fisher). Supernatant containing lentivirus particles was generated from HEK293T cells cotransfected with expression vector and packaging plasmids pMD2.G and psPAX2 using Fugene 6 (Promega). Supernatants were filtered through a 0.45-µm filter and added to primary fibroblasts from P1 cultured in 6-well plates. The transduction efficiency of fibroblasts based on expression of EmGFP was ∼70%.

### Phosflow STAT3 and STAT1 signaling assay

Unless indicated otherwise, phosphorylation of STAT1 or STAT3 transcription factors was determined by Phosflow after 15 min of cytokine stimulation. Analysis of primary blood lymphocytes and monocytes was performed in whole blood. Cells were stained with different combinations of the following antibodies: anti-CD19–FITC (clone HIB19; Biolegend), anti-CD19–BV711 (clone HIB19; Biolegend), anti-CD14–BV650 (clone M5E2; Biolegend), anti-CD3–BV711 (clone UCHT1; BD biosciences), and anti-CD3–PE-Cy5 (clone UCHT1; Biolegend). Cells were subsequently activated with 100 ng/ml IL-6, 50 ng/ml IL-10, 100 ng/ml IL-21, or 100 ng/ml IL-27 before immediate fixation and lysis of red blood cells (BD biosciences). For analysis of STAT3 phosphorylation in T lymphoblasts, cells were extensively washed and rested in complete medium without IL-2 for 2 h before activation with 100 ng/ml IL-6, 50 ng/ml IL-10, 100 ng/ml IL-21, or 100 ng/ml IL-27. Levels of phospho-STAT3 in LCLs were determined in serum-starved cells, stimulated with 50 ng/ml IL-6 or 50 ng/ml IL-21 before resuspending cells in Cytofix buffer (BD biosciences). To determine the effect of different concentrations of GP130-family cytokines, HEK293 GP130-KO cells were plated in 24-well plates and transiently transfected with empty plasmid or plasmid encoding WT or mutant GP130 using Lipofectamine 2000 (Thermo Fisher). Cells were cotransfected with IL-6RA or IL-11RA to enhance phosphorylation signal and with GFP to allow gating on successfully transfected cells. 24 h after transfection, cells were serum starved for 2 h, followed by stimulation with the indicated cytokines at different concentrations and immediate detachment with trypsin-EDTA solution and fixation in Cytofix buffer. Similarly, fibroblasts plated in 12-well plates were stimulated with IL-6–family cytokines at different concentrations, immediately detached with trypsin-EDTA solution, and resuspended in Cytofix buffer. After fixation, primary cells, LCLs, and cell lines were washed in PBS, permeabilized with ice-cold Perm Buffer III (BD biosciences), and stained with anti-STAT3 (pY705)–Alexa Fluor 647 (clone 4/P-STAT3) and anti-STAT1 (pY701)–BV421 or Alexa Fluor 488 (clone 4a; both BD biosciences) at room temperature. Samples were acquired on an LSRII or LSRFortessa flow cytometry system, and data were analyzed with FlowJo software (version 10.0.6).

### Immunofluorescence and confocal microscopy

HEK293 GP130-KO cells were seeded on poly-l-lysine–coated 8-well chamber slides (Sarstedt) and transiently transfected with empty control plasmid or plasmid encoding WT or mutant GP130 using Lipofectamine 3000 (Thermo Fisher). The next day, cells were rested in serum-free medium for 1 h and activated with 0.5 ng/ml IL-6 for 15 min. After fixation with 2% paraformaldehyde, cells were permeabilized with ice-cold methanol for 10 min before staining with anti-STAT3 (no. 12640; Cell Signaling) and appropriate secondary antibody (Alexa Fluor 568 goat anti–rabbit IgG; Life Technologies). Cell nuclei were counterstained with DAPI, and slides were mounted with Vectashield mounting medium (Vector Laboratories). Images were acquired with an inverted confocal microscope (510; ZEN2009 software; Zeiss) and analyzed with ImageJ software (National Institutes of Health; Schindelin et al., 2012). For illustration of STAT3 nuclear translocation in HEK293 cells, images were processed by Photoshop (CS4; Adobe).

### Luciferase STAT3 reporter assay

The dual-luciferase STAT3 reporter assay was obtained from Qiagen. HEK293 GP130-KO cells were transiently transfected with WT or mutant GP130-expressing plasmids in multiple replicates of an opaque white 96-well plate with Lipofectamine 3000 following the manufacturer’s protocol. To assess cytokine signaling, HEK293 GP130-KO cells were cotransfected with IL-6RA and IL-11RA expression vectors. After 24 h, cells were rested in serum-free medium for 2 h followed by stimulation with 0.5 ng/ml recombinant human IL-6 or 1 ng/ml IL-11 for 6 h. Cells were lysed directly in opaque 96-well plates, and luminescent signal from Firefly and Renilla reporter genes was quantified with the Dual-Glo Luciferase Assay system (Promega) according to the manufacturer’s instructions on a FLUOstar OPTIMA plate reader (BMG labtech). Data were normalized to the activity of Renilla luciferase.

### Quantitative PCR mRNA expression

Quantitative PCR was performed according to established standard protocols. Total RNA was extracted from cells using the RNeasy mini kit (Qiagen), and cDNA was synthesized using the High Capacity cDNA Reverse Transcription kit (Applied Biosystems). Gene expression was determined using Taqman assays from Applied Biosystems: *fibrinogen α chain* (Hs00241027_m1), *fibrinogen β chain* (Hs00170586_m1), and *RPLP0* (Hs99999902_m1). For induction of acute phase genes, Hep3B GP130-KO cells were stimulated with 50 ng/ml IL-6 for 24 h in complete medium. Transient transfection with GP130 WT or variant plasmid or empty vector was performed 24 h before stimulation using Lipofectamine 2000. Results were normalized to the housekeeping gene *RPLP0* and expressed as fold-induction compared with the unstimulated condition.

### Modeling of mutations

All protein structures were analyzed and rendered in PyMol (version 1.4; DeLano Scientific). Coordinate files were obtained from the Protein Data Bank (PDB; accession no. 1P9M for IL-6/IL-6RA/GP130; accession no. 3L5H for GP130 D1-D6).

### Protein sequence alignment

Multiple sequences were aligned using ClustalW2 (Goujon et al., 2010). Data were obtained from NCBI. Sequence alignment is based on the following RefSeq accession nos.: NP_001106976.1, NP_001124412.1, NP_990202.1, NP_034690.3, NP_002175.2, NP_004834.1, NP_000751.1, NP_002301.1, NP_003990.1, NP_005526.1, and NP_001550.1.

### Statistical analysis

Results were analyzed with Prism (version 5.00; GraphPad software, Inc.). Significance of differences was determined by two-sided Mann-Whitney *U* tests, and p-values <0.05 were considered significant.

### Online resources

The following online data sources have been accessed: 1000 Genomes, dbSNP, GenBank, gnomAD, PolyPhen, SIFT, STRING, ExAC browser, GDI-server, and COSMIC.

### Online supplemental material

Fig. S1 shows the generation and functional consequences of CRISPR/Cas9-mediated GP130-KO. Fig. S2 shows structural analysis of the GP130 p.N404Y substitution. Table S1 lists homozygous nonsynonymous variants in P1 identified by exome sequencing.

## Supplementary Material

Supplemental Materials (PDF)
