# Supplementary material for: A biallelic mutation in IL6ST encoding the GP130 co-receptor causes immunodeficiency and craniosynostosis
Source: J Exp Med. 2017 Sep 4;214(9):2547–62. doi: 10.1084/jem.20161810 (PMC5584118; doi:10.1084/jem.20161810)
Supplement: Supplemental Materials (PDF) [file JEM_20161810_sm.pdf]

SUPPLEMENTAL MATERIAL

Schwerd et al., https://doi.org/10.1084/jem.20161810

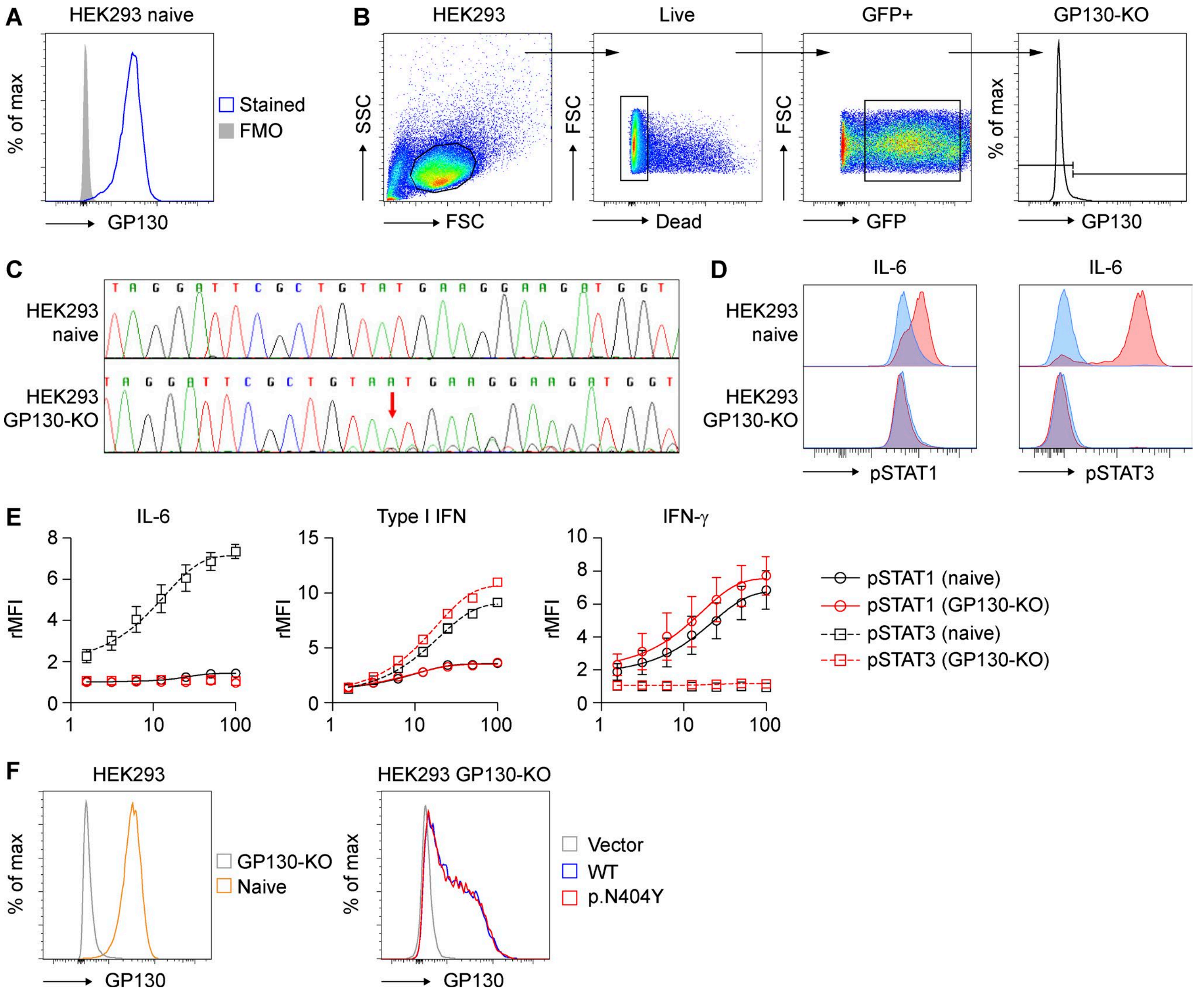

Figure S1. **CRISPR/Cas9-mediated GP130-KO abrogates IL-6 signaling and does not affect signaling of GP130-independent cytokines.** (A) Naive HEK293 cells express GP130. FMO, fluorescence minus one. (B) Loss of GP130 expression in CRISPR/Cas9-transfected HEK293 cells. HEK293 cells were transfected with plasmid encoding CRISPR/Cas9, a GFP reporter, and gRNA directed against GP130. Live GFP+ cells were analyzed for GP130 expression 6 d after transfection. FSC, forward scatter; SSC, side scatter. (C) Dideoxy sequencing confirms CRISPR/Cas9-mediated gene disruption by a homozygous frame-shift insertion of A (red arrow) in *IL6ST* exon 8. (D) Functional validation of the GP130-KO cell line using flow cytometry analysis of phospho-STAT1 (pSTAT1) and phospho-STAT3 (pSTAT3). Naive and GP130-KO cells were stimulated with 10 ng/ml IL-6 for 15 min. (E) Stimulation of the HEK293 parent cell line and HEK293 GP130-KO cells with GP130-independet cytokines at the indicated concentrations (ng/ml) results in comparable levels of pSTAT1 and pSTAT3. WT and GP130-KO cells were stimulated with type I IFN and IFN-γ for 15 min, and STAT1/3 phosphorylation was assessed by flow cytometry. Stimulation with IL-6, a GP130-dependent cytokine, shows abrogated signaling in the GP130 KO cell line. Stimulation of HEK293 cells with FGF, EGF (epidermal growth factor), IL-10, and IL-22 did not result in STAT1 or STAT3 phosphorylation, in neither WT cells nor in GP130-KO cells (not depicted). Data represent mean with SEM. Results are based on four (type I IFN), five (IFN-γ), and six (IL-6) replicates from four independent experiemnts. rMFI, relative mean fluorescence intensity. (F) Transient overexpression of GP130 WT and mutants in HEK293 GP130-KO cells results in comparable surface expression. (Left) GP130 gene disruption by CRISPR/Cas9 in HEK293 cells results in absence of GP130 surface expression. (Right) Flow cytometric analysis of HEK293 GP130-KO cells transfected with empty vector control or plasmids encoding GP130 WT or the patient variant p.N404Y. Cells were stained for GP130 surface expression 24 h after transfection. Results are representative of three independent experiments. Compared with 24 h, transfection for 48 h results in overall decreased GP130 expression but still equal levels between WT and p.N404Y (not depicted). All experiments with transient overexpression of GP130 were performed after 24 h.

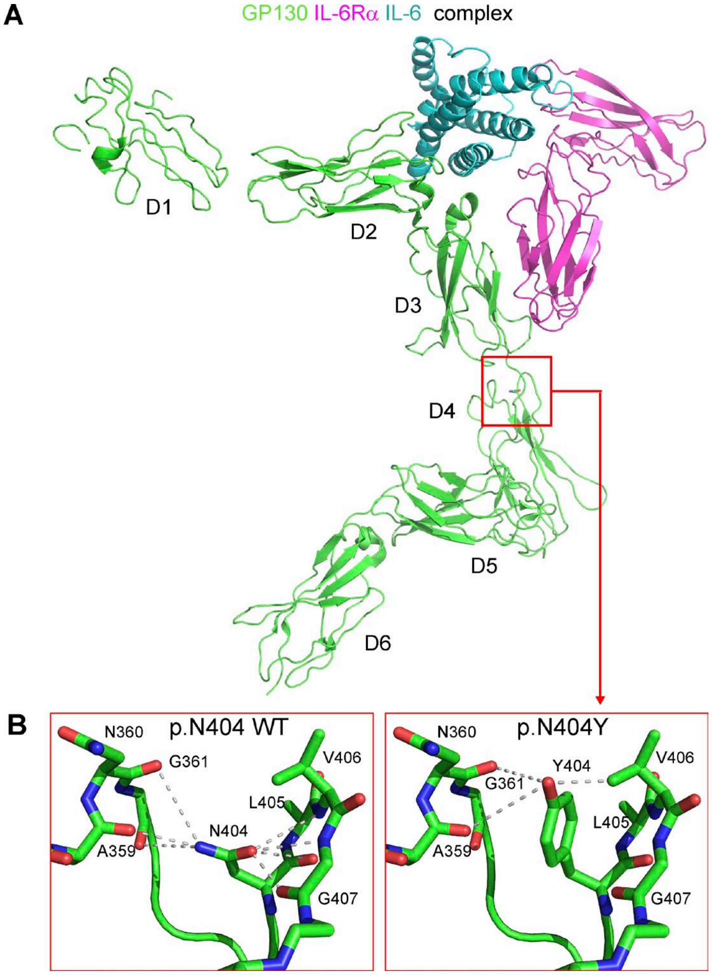

Figure S2. **Structural analysis of the IL-6ST substitution.** (A) Model of the IL-6/IL-6RA/GP130 D1–D6 tripartite complex in cartoon representation, obtained from an overlay of the published IL-6/IL-6RA/GP130 D1–D3 crystal structure (Boulanger et al., 2003; PDB accession no. 1P9M) onto the respective domains of the GP130 D1–D6 crystal structure of the full ectodomain (Xu et al., 2010; PDB accession no. 3L5H). The GP130 molecule is colored in light green, the IL-6RA in magenta, and the IL-6 in cyan. (B) Cartoon and sticks illustration of the GP130 D4 β-turn containing either p.N404 or the p.N404Y substitution. Dotted lines indicate potential intramolecular hydrogen bonding networks.

Table S1. **Likely homozygous nonsynonymous variants in P1 identified by exome sequencing**

Gene	Nucleotide and amino acid change	ESP6500[a]	1000g2012[b]	dbSNP137[c]	Del Score[d]	CADD score[d]	Chr	Position
IL6ST	NM_001190981:c.1210A>T:p.N404Y				6	27.2	5	55251910
MED15	NM_015889:c.2132C>T:p.S711L				5	23.1	22	20940876
MFSD6L	NM_152599:c.1720G>A:p.D574N	0.007919	0.0037	rs78093130	5	32	17	8700719
SEMA5B	NM_001256348:c.1127A>G:p.N376S				5	20.8	3	122641266
LAMA1	NM_005559:c.3838G>A:p.G1280R				5	25.4	18	7010234
NUDT9	NM_001248011:c.416C>A:p.A139E				5	32	4	88363049
ARHGAP24	NM_031305:c.1774G>A:p.D592N				4	29.3	4	86921681
COL10A1	NM_000493:c.382G>A:p.D128N	0.000692	0.0023	rs142463796	4	17.21	6	116442897
CHST6	NM_021615:c.993G>T:p.Q331H	0.001231	0	rs140699573	4	27.4	16	75512734
TDRD5	NM_001199085:c.151C>T:p.R51W	0.000077	0.0009	rs138668107	4	32	1	179561901
OSBPL10	NM_001174060:c.364C>G:p.L122V				4	23	3	31921240
TNN	NM_022093:c.2062G>T:p.V688L	0.000154	0.0005	rs149504279	3	25.7	1	175067674
SENP5	NM_152699:c.1340C>A:p.S447Y			rs202085742	3	22.5	3	196613392
SMG7	NM_001174061:c.591G>C:p.E197D				3	27.2	1	183498542
IRS1	NM_005544:c.3304G>A:p.E1102K				3	25.7	2	227660151
ZNF451	NM_001031623:c.2261G>A:p.R754H				3	23.8	6	57013144
CHIT1	NM_001256125:c.119G>A:p.R40H	0.009842	0.02	rs35920428	3	23.6	1	203194935
TDRD6	NM_001010870:c.3230A>G:p.D1077G	0.000077		rs141532119	3	23.7	6	46659095
SF3B2	NM_006842:c.151G>A:p.V51M				3	29.4	11	65820161
SCFD2	NM_152540:c.938C>T:p.A313V	0.001768	0.0005	rs147606542	2	13.64	4	54218834
CHIT1	NM_001256125:c.707C>G:p.T236S	0.008765	0.02	rs61745299	2	18.76	1	203188943
SMC1B	NM_148674:c.2874A>C:p.E958D			rs199833095	2	16.59	22	45754664
WDR4	NM_001260474:c.70G>T:p.A24S	0.000154	0.0005	rs150449975	2	32	21	44299536
LRRC37A	NM_014834:c.3423C>A:p.F1141L			rs1863115	2	21.3	17	44408066
NPSR1	NM_207172:c.380T>C:p.L127S				2	25.1	7	34818173
CORO1B	NM_020441:c.650C>G:p.A217G				2	9.17	11	67208702
ABL2	NM_001136000:c.70C>A:p.L24M			rs202125612	2	7.39	1	179112110
DOCK8[e]	NM_001190458:c.459C>A:p.D153E	0.002384		rs139391329	1	5.293	9	312088
ZSCAN16	NM_025231:c.989G>A:p.R330Q				1	24.8	6	28097670
PPME1	NM_016147:c.215G>A:p.S72N	0.000084			1	22.4	11	73915417
DST	NM_001723:c.6007G>A:p.A2003T		0.0005	rs74874398	1	8.23	6	56482825
TBC1D10A	NM_001204240:c.1433C>T:p.P478L	0.000231		rs201271557	1	0.246	22	30688479
CTRB1	NM_001906:c.37G>A:p.V13M				1	6.79	16	75252942
KIAA0040	NM_001162895:c.31A>G:p.I11V	0.012046	0.01	rs116333252	0	0.032	1	175130119
MFSD6L	NM_152599:c.625G>A:p.A209T	0.00246	0.0009	rs144238966	0	3.486	17	8701814
EYS	NM_001142800:c.8422G>A:p.A2808T	0.00657	0.01	rs111991705	0	13.78	6	64431505
MFSD6L	NM_152599:c.1195G>A:p.G399S	0.002691	0.0032	rs138080986	0	0.031	17	8701244
EFCAB12	NM_207307:c.949G>A:p.D317N	0.013588	0.04	rs75410160	0	8.242	3	129130087
TWISTNB	NM_001002926:c.752C>G:p.A251G				0	10.24	7	19738204
MFSD6L	NM_152599:c.1124C>T:p.T375I	0.002768	0.0009	rs111767864	0	4.83	17	8701315
AHNAK2	NM_138420:c.2239C>A:p.P747T		0.01	rs117125675	0	0.004	14	105419549
AHNAK2	NM_138420:c.2178G>C:p.Q726H	0.000164	0.01	rs117226478	0	22.7	14	105419610
MUC4	NM_018406:c.12160G>A:p.A4054T				0	0.15	3	195506291
SAG	NM_000541:c.511A>G:p.K171E				0	21.9	2	234235842
KHDC1L	NM_001126063:c.47T>C:p.L16P				0	22.5	6	73935085
PLA2G7	NM_001168357:c.545G>T:p.R182I	0.000077		rs146433848	0	11.72	6	46679351
DNAH11	NM_001277115:c.11756C>T:p.T3919I				0	-	7	21907521
LRRC37A	NM_014834:c.4612A>G:p.K1538E			rs62073249	0	11.2	17	44409255
ANKRD6	NM_001242814:c.263C>T:p.T88M				0	22.2	6	90312791

[a]ESP6500, variant frequency from the NHLBI Exome Sequencing Project.
[b]1000g2012, variant frequency from the 1000 Genomes Project.
[c]dbSNP137, annotation in build 137 of dbSNP.
[d]The severity of amino acid substitutions (Del = deleterious) was predicted using a combination of the following six metrics: Polyphen2, SIFT, PhyloP, LRT, Mutation Taster, and GERP++ (Fu et al., 2013), or using combined annotation–dependent depletion (CADD) scores (Kircher et al., 2014).
[e]This *DOCK8* variant was considered unlikely to be a major contributor to the phenotype of P1 for the following reasons: (a) The clinical phenotype of P1 is not typical for DOCK8-HIES. P1 presented with four of the five criteria that are typical for STAT3 signaling defects (lung abnormalities, eosinophilia, upper respiratory infections, and retained primary teeth) but not DOCK8 (Engelhardt et al., 2015). (b) The *DOCK8* variant is found at an allele frequency of ∼0.2% in non-Finnish Europeans and the South Asian population, yet is not described as pathogenic in the DOCK8 literature (Engelhardt et al., 2015). (c) The low del and CADD scores of the *DOCK8* variant are not strongly suggestive of pathogenicity. (d) As described here, the STAT3-associated cytokine signaling defect in P1 was rescued by WT GP130 transfection, confirming the causal role of the *IL6ST* variant.

REFERENCES

Boulanger, M.J., D.C. Chow, E.E. Brevnova, and K.C. Garcia. 2003. Hexameric structure and assembly of the interleukin-6/IL-6 α-receptor/gp130 complex. *Science.* 300:2101–2104. http://dx.doi.org/10.1126/science.1083901

Engelhardt, K.R., M.E. Gertz, S. Keles, A.A. Schäffer, E.C. Sigmund, C. Glocker, S. Saghafi, Z. Pourpak, R. Ceja, A. Sassi, et al. 2015. The extended clinical phenotype of 64 patients with dedicator of cytokinesis 8 deficiency. *J. Allergy Clin. Immunol.* 136:402–412. http://dx.doi.org/10.1016/j.jaci.2014.12.1945

Fu, W., T.D. O'Connor, G. Jun, H.M. Kang, G. Abecasis, S.M. Leal, S. Gabriel, M.J. Rieder, D. Altshuler, J. Shendure, et al. NHLBI Exome Sequencing Project. 2013. Analysis of 6,515 exomes reveals the recent origin of most human protein-coding variants. *Nature.* 493:216–220. http://dx.doi.org/10.1038/nature11690

Kircher, M., D.M. Witten, P. Jain, B.J. O'Roak, G.M. Cooper, and J. Shendure. 2014. A general framework for estimating the relative pathogenicity of human genetic variants. *Nat. Genet.* 46:310–315. http://dx.doi.org/10.1038/ng.2892

Xu, Y., N.J. Kershaw, C.S. Luo, P. Soo, M.J. Pocock, P.E. Czabotar, D.J. Hilton, N.A. Nicola, T.P. Garrett, and J.G. Zhang. 2010. Crystal structure of the entire ectodomain of gp130: insights into the molecular assembly of the tall cytokine receptor complexes. *J. Biol. Chem.* 285:21214–21218. http://dx.doi.org/10.1074/jbc.C110.129502
